# First person – Kelly Diamond

**DOI:** 10.1242/bio.059235

**Published:** 2022-02-17

## Abstract

First Person is a series of interviews with the first authors of a selection of papers published in Biology Open, helping early-career researchers promote themselves alongside their papers. Kelly Diamond is first author on ‘ Computational anatomy and geometric shape analysis enables analysis of complex craniofacial phenotypes in zebrafish’, published in BiO. Kelly is a postdoc in the lab of Dr Murat Maga at the Center for Developmental Biology and Regenerative Medicine, Seattle Children's Research Institute, Seattle, USA, investigating which combination of factors contribute to organismal morphology.


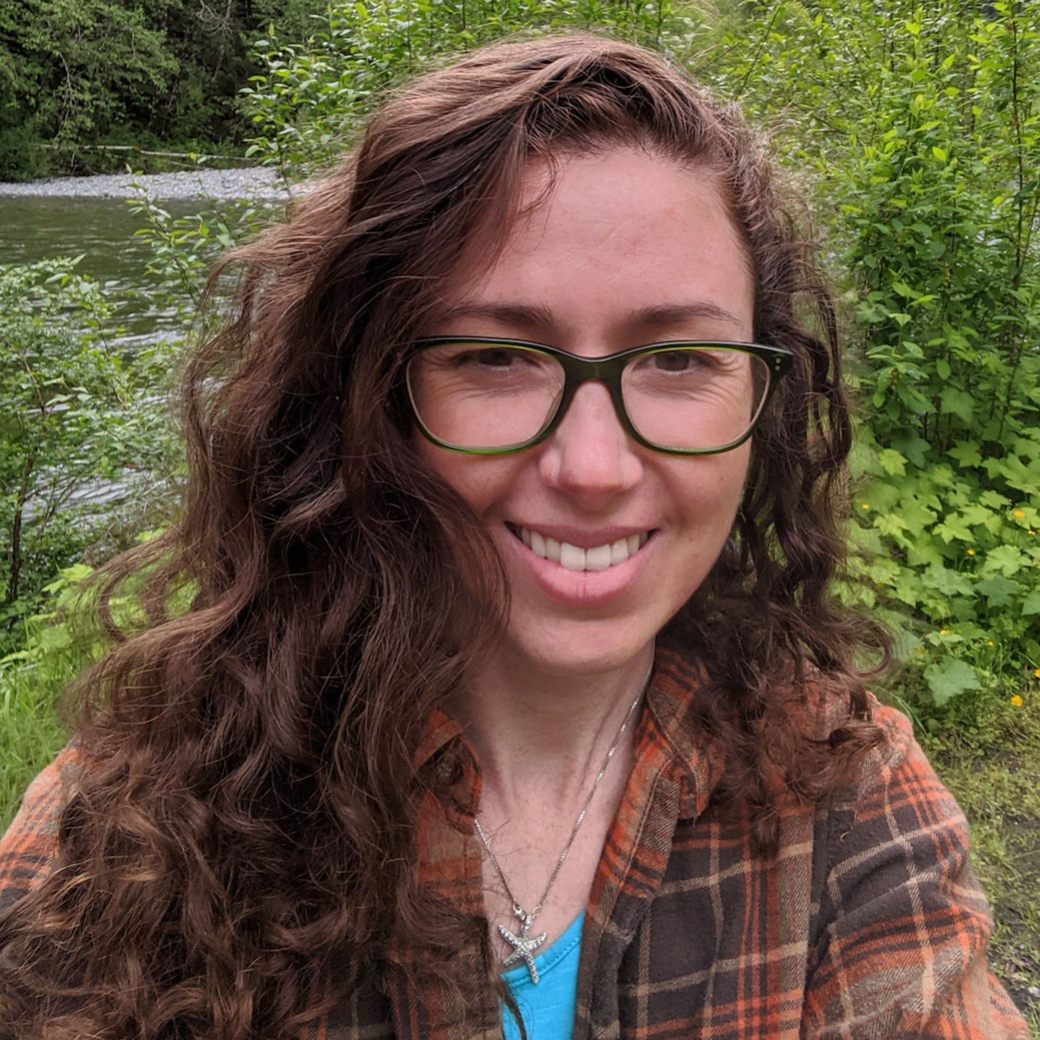


**Kelly Diamond**

**What is your scientific background and the general focus of your lab?**

I use an integrative approach to examine how both genetic and ecological mechanisms drive the functional morphology observed in nature. My research is inspired by the natural history of organisms and integrates AI methods, such as machine learning and computational anatomy, with biomechanics and statistics to explore patterns in ecomorphology and organismal performance. My graduate work focused on form-function relationships in a group of amphidromous goby fishes. Currently I am developing semi-automated methods to phenotype the zebrafish craniofacial skeleton.

**How would you explain the main findings of your paper to non-scientific family and friends?**

Many of the same or similar genes that have been linked to human skeletal diseases, are also found in zebrafish. We developed a method for identifying how specific genes alter skull shape. Using a gene that has been linked to brittle bone disease in humans as an example, we found three regions of the skull that differed between zebrafish that had a working gene and those that did not.

**What are the potential implications of these results for your field of research?**

Our approach offers a potential pipeline for high-throughput screening of complex fish craniofacial shape to discover novel phenotypes for zebrafish, and important model system for developmental biology, evolution, and human disease.

**Figure:**
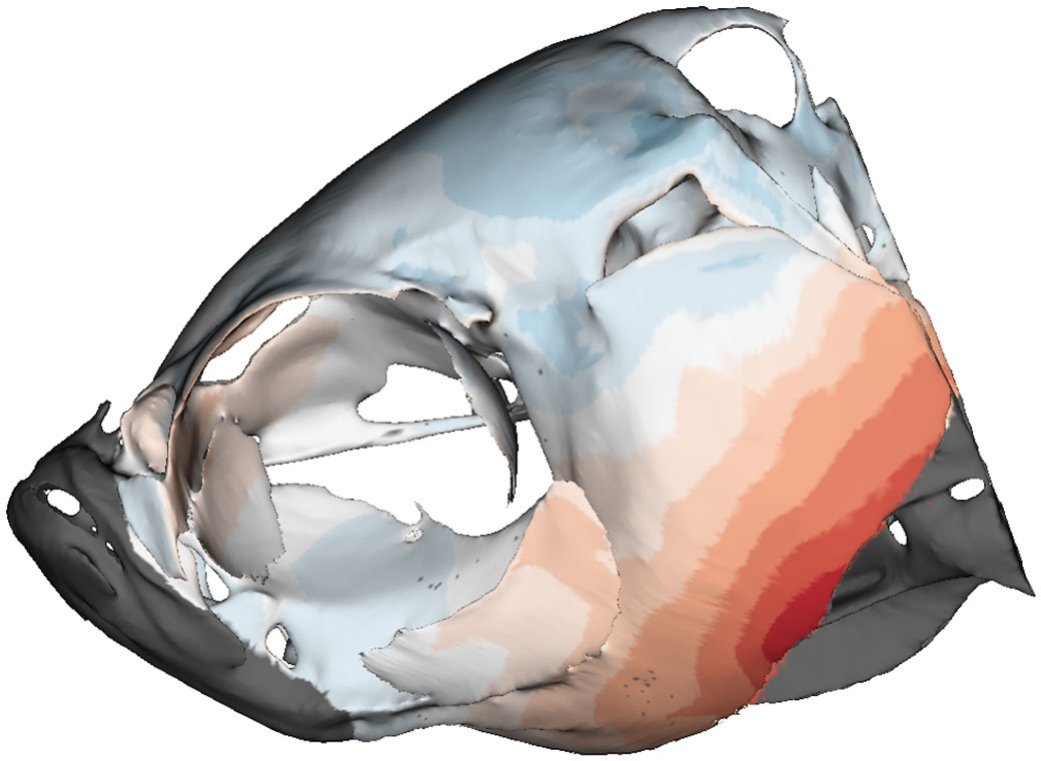
Heatmap showing differences between mean shapes of wild-type and crispant zebrafish craniofacial skeletons.

**What has surprised you the most while conducting your research?**

One of the first things we found in this project was that the structures in the ear that help fish with their hearing and vestibular function, the otoliths, were larger in the *bmp1a* crispant fish than the wild-type controls. We are not sure of the mechanism yet, but it was an unexpected finding.

**What, in your opinion, are some of the greatest achievements in your field and how has this influenced your research?**

The greatest achievement in quantitative morphology is the adoption of using open-source tools to build a community of researchers and developers working toward common goals. Tools such as the ANTs ecosystem and 3D Slicer, that we use in this study have allowed for fast and efficient development of cutting-edge methods and pipelines.

**What changes do you think could improve the professional lives of early-career scientists?**

Postdocs in particular need more stability by offering longer contracts and more flexible timelines. It is difficult to focus on a project when you have to spend so much time and energy searching and applying for your next job.

**What's next for you?**

Currently I am working with a team of undergraduates from the University of Washington to expand the application of the pipeline presented in this paper to a broader range of target genes. I will be starting as an Assistant Professor of Biology at Rhodes College in the Fall of 2022 where I will continue my current quantitative morphology collaborations while also starting projects that have more of an ecological focus.

## References

[BIO059235C1] Diamond, K. M., Rolfe, S. M., Kwon, R. Y. and Maga, A. M. (2022). Computational anatomy and geometric shape analysis enables analysis of complex craniofacial phenotypes in zebrafish. *Biol. Open* 11, bio058948. 10.1242/bio.058948 35072203 PMC8864294

